# Post-stroke infections associated with spleen volume reduction: A pilot study

**DOI:** 10.1371/journal.pone.0232497

**Published:** 2020-05-11

**Authors:** Amber Nous, Ilse Peeters, Koenraad Nieboer, Anne-Marie Vanbinst, Jacques De Keyser, Sylvie De Raedt

**Affiliations:** 1 Department of Neurology, Universitair Ziekenhuis Brussel, Center for Neurosciences, Vrije Universiteit Brussel, Brussels, Belgium; 2 Department of Radiology, Universitair Ziekenhuis Brussel, Vrije Universiteit Brussel, Brussels, Belgium; Fiji national University School of Medicine, FIJI

## Abstract

**Background:**

Spleen volume reduction followed by re-expansion has been described in acute ischemic stroke in both animal and human studies. Splenic contraction might be partially due to sympathetic hyperactivity and might be accompanied by release of splenocytes in the peripheral circulation, leading to immunodepression.

**Aims:**

To investigate whether spleen volume changes in the first week after stroke are associated with post-stroke infections, changes in lymphocytes count and autonomic dysfunction.

**Methods:**

In patients with acute ischemic stroke, spleen sizes were calculated from abdominal CT images on day one and day seven. Spleen size reduction was defined as > 10% spleen size reduction between day one and day seven. Post stroke infections were diagnosed during the first seven days after stroke onset using the modified criteria of the US Center of Disease Control and Prevention. We assessed the time course of leukocyte subsets and analysed pulse rate variability (PRV) indices.

**Results:**

Post-stroke infections occurred in six out of 11 patients (55%) with spleen size reduction versus in five out of 27 patients (19%) without spleen size reduction (p = 0,047). Spleen size reduction was associated with a drop in lymphocytes and several lymphocyte subsets from admission to day one, and a higher NIHSS at admission and at day three (p = 0,028 and p = 0,006 respectively). No correlations could be found between spleen volume change and PRV parameters.

**Conclusion:**

Post-stroke infections and a drop in lymphocytes and several lymphocyte subsets are associated with spleen volume reduction in acute ischemic stroke.

## Introduction

Growing evidence supports the role of the spleen in post-stroke immunosuppression. Both animal and human studies showed splenic contraction the first 24–48 hours in acute ischemic stroke, mostly followed by a re-expansion. [1–4] A persistent splenic contraction after 48 hours might be associated with poor clinical outcome. [3] Splenic contraction is associated with a dramatic reduction of the number of immune cells in the spleen (especially lymphocytes), probably explained by increased cell death and release of cells into the blood circulation and migration to the brain. [1,4–8] After an initial pro-inflammatory phase, the reduction of immune cells results in an immunosuppressed status which increases the susceptibility to post-stroke infections. [1,6,9] Autonomic dysfunction, particularly sympathetic hyperactivity [10] appears to play a crucial role in the development of stroke-induced immune depression. There is evidence that splenic innervation is predominantly sympathetic and that catecholamines can induce splenic atrophy. [1,6,9] On the other hand, blockade of α- and β-adrenergic receptors inhibits shrinkage of the spleen. [1] So, autonomic dysfunction might be one of the mechanisms that can explain splenic contraction.

The aim of this study was to investigate whether post-stroke infections, lymphocyte-subset changes, autonomic parameters and functional outcome are different in patients with versus without spleen volume reduction during the first week after acute ischemic stroke.

## Methods

### Subjects

38 patients with acute ischemic stroke, admitted within eight hours after symptom onset or after wake-up symptoms, were prospectively included in the study. Exclusion criteria were: haematological disorders with effect on white blood cell counts, infections preceding stroke (last week before stroke) based on clinical investigation, CRP and temperature during the first examination, use of immunosuppressive medication, malignancy with active treatment (chemo- or radiotherapy), use of antibiotics 24 hours before stroke, auto-immune diseases, pregnancy and age under 18 years old.

Ischemic stroke was diagnosed clinically, confirmed by cerebral CT upon admission and/or brain MRI within the first 10 days after stoke. MRI was performed between admission and day 10 on a 1.5 T (Philips) or 3T (Siemens) MRI scanner, including T1, T2, fluid attenuated inversion recovery (FLAIR) and diffusion weighted imaging (DWI). Infarction volume was calculated on the DWI sequences with dedicated software from Olea Medical® (Marseille, France). Stroke severity was measured by certified neurologists using the National Institute of Health Stroke Score (NIHSS) on admission, days one and three. Poor functional outcome was defined as a modified Rankin Scale (mRS) score of > 2 at three months. Early neurological deterioration was defined as an increase in NIHSS by 2 or more points or stroke-related death between admission and day three.

The protocol was approved by the local Ethics Committee (University Hospital of Brussels). All patients or their next-on-kin signed a written informed consent.

### Study procedures

#### 1. CT-scan upper abdomen

The volume of the spleen was calculated using unenhanced CT images of the upper abdomen, acquired on day one (between 24 and 48 hours after stroke) and day seven (between 144 hours and 168 hours after stroke). Spleen images were transferred to a dedicated workstation (AW workstation VolumeShare 7, General Electric Healthcare, Milwaukee USA or Extended Brilliance Workspace V4.5, Royal Philips NV, Amsterdam, The Netherlands) on which spleen volumes were segmented and calculated in volume rendering. The entire volume in cm³ was displayed after acceptance by the operator. Spleen volume reduction was defined as a > 10% decrease in spleen size between days one and seven after stroke onset.

#### 2. Blood samples

Blood samples were taken on admission and day 1 (between 24 to 48 hours after stroke). White blood cells, neutrophils, monocytes, lymphocytes and lymphocyte subsets were determined. Neutrophils, monocytes and lymphocytes were counted in peripheral blood by use of fluorescent flowcytometric measurements (CELL-DYN Sapphire, Abbott Diagnostics, Abbott Park, IL). Lymphocyte subsets were counted in peripheral blood by use of monoclonal antibodies (FC500 flow cytometer, Beckman Coulter). The following antibodies were used: T cells (CD3+), T helper cells (CD4+), cytotoxic T cells (CD8+), B cells (CD19+) and Natural Killer (NK) cells (CD3−CD56+CD16+). White blood cells, neutrophils, monocytes and lymphocytes were also determined on day seven to further analyse their evolution.

#### 3. Infections

Infections were diagnosed during the first seven days after stroke onset using the modified criteria of the US Center for Disease Control and Prevention 2012. Pneumonia was diagnosed when at least one of the former and one of the latter criteria were fulfilled: (1) abnormal respiratory examination, pulmonary infiltrates on chest x-rays; (2) productive cough with purulent sputum, microbiological cultures from lower respiratory tract or blood cultures, leukocytosis, elevation of C-reactive protein (CRP). Urinary tract infection was diagnosed when at least two of the following criteria were present: fever (>38°C), urine sample positive for nitrite, leukocyturia, and significant bacteriuria. [11]

#### 4. Autonomic dysfunction

Pulse rate variability (PRV) indices were calculated from 5 minute beat-to-beat blood pressure recordings obtained via a Nexfin® monitor (BMEYE B.V., Amsterdam, The Netherlands) within the first 24 hours after stroke onset during a resting period. Patients with atrial fibrillation were excluded. The Nexfin® device measures non-invasive beat-to-beat arterial blood pressure by the volume clamp method through plethysmography using an inflatable finger cuff. PRV derived from non-invasive blood pressure waveforms corresponds sufficiently with traditional heart rate variability (HRV) derived from ECG in subjects at rest. [12] PRV was calculated using Kubios software (University of Eastern Finland, Kuopio/Finland). We calculated the most commonly used time domain and spectrum domain HRV parameters to estimate PRV on 5 minute recordings, which were standard deviation of a series of RR intervals (SDNN), root mean square differences of successive RR intervals (RMSSD), high frequency range (HF), low frequency range (LF) and LF/HF.

### Statistical analysis

Values are reported as number or median with interquartile range (IQR), displayed as Q1 and Q3.

The Fisher Exact Test (FE) and the Mann-Whitney U test (MWU) were applied to detect statistically significant differences in respectively categorical binary data (FE) and categorical ordinal or continuous (MWU) variables between the groups with versus without splenic contraction. The Wilcoxon Signed Ranks Test or Friedman Test with Bonferroni correction were used to detect differences in variables within the same group.

Because of low sample size, data were considered as not normally distributed.

Correlations were calculated with the Spearman Rank correlation coefficient.

Analyses were performed using the SPSS®25.0 software package and GraphPad Prism®8.0. A P-value of < 0.05 was considered significant.

## Results

### Spleen volumes

Median spleen volume was 160 cm³ [IQR 133;201] on day one and 159 cm³ [IQR 130;202] on day seven. Eleven patients (29%) showed a spleen volume reduction of > 10% between day one and day seven. These patients showed a median reduction of -33 cm³ [IQR -41;-30] from day one to day seven, or a median reduction of -17% [IQR -21%;-16%].

### Main characteristics of patients with and without splenic contraction

Main characteristics of patients with and without splenic contraction are shown in Table 1.

**Table 1:** Main characteristics of patients with and without spleen volume reduction.

	Spleen Volume Reduction (n = 11)	No Spleen Volume Reduction (n = 27)	P-value
**Baseline characteristics**			
Female Sex	4 [36%]	9 [33%]	1
Age, yrs	70 [IQR 59;85]	77 [IQR 65;82]	0,974
**Stroke characteristics**			
Stroke Volume, cm^3^	4 [IQR 3;42]	3 [IQR 1;14]	0,161
Thrombolysis	9 [81%]	17 [63%]	0,444
NIHSS–admission	11 [IQR 6;17]	7 [IQR 5;9]	**0,028**
NIHSS—day 1	10 [IQR 4;15]	5 [IQR 2;6]	0,075
NIHSS—day 3	10 [IQR 4;17]	2 [IQR 1;5]	**0,006**
**Outcome**			
Early neurological deterioration	2 [18%]	0 [0%]	0,078
Poor neurological outcome	8 [73%]	9 [33%]	0,141
Mortality	3 [27%]	2 [7%]	0,300
Post-stroke infection	6 [55%]	5 [19%]	**0,047**
**PRV parameters**			
SDNNrr	21 [IQR 19;25]	26 [IQR 14;37]	0,809
RMSSD	20 [IQR 14;32]	25 [IQR 15;31]	0,717
HFms^2^	134 [IQR 27;246]	149 [IQR 48;580]	0,468
HF%	34 [IQR 26;39]	40 [IQR 24;44]	0,545
LFms^2^	39 [IQR 24;78]	129 [IQR 31;302]	0,304
LF%	16 [IQR 9;50]	32 [IQR 20;50]	0,431
LF/HF	0,7 [IQR 0,3;1,8]	1 [IQR 0,63;1,24]	0,628
**Beta-blocker use**			
Prior to stroke	2 [18%]	10 [37%]	0,444
During hospitalisation	3 [27%]	9 [33%]	1,000

Data are expressed as median +/- IQR [Q1,Q3] or number as appropriate. P-values were calculated with the Fisher Exact test and Mann-Whitney U test. Poor neurological outcome data were missing in 4 patients, PRV parameters in 13 patients due to atrial fibrillation, pacemaker, technical problems with the Nexfin monitor.

Abbreviations: NIHSS = National Institutes of Health Stroke Scale, PRV = pulse rate variability, SDNN = standard deviations of all NN-intervals, RMSSD = the square root of the mean of the sums of the squares of differences between adjacent NN intervals, LF = low frequency power, HF = high frequency power, ms^2^ = absolute power; % = relative power = absolute power (ms^2^)/total power (ms^2^)*100%.

Post-stroke infections occurred in six out of 11 (55%) patients with splenic contraction, and in five out of 27 (19%) patients without splenic contraction (p = 0,047). In the group with splenic contraction, three patients developed pneumonia and three patients urinary tract infection (UTI). In the group without splenic contraction, four patients developed UTI and 1 patient developed pneumonia. Timing of infection was similar in both groups, varying from day one to day five after stroke. Patients with spleen volume reduction had higher NIHSS on admission and at day three than those without spleen volume reduction. PRV parameters were similar in patients with and without spleen volume reduction. No differences in β-blocker use could be found between both groups.

### Lymphocytes, monocytes and neutrophils

**Fig 1:**
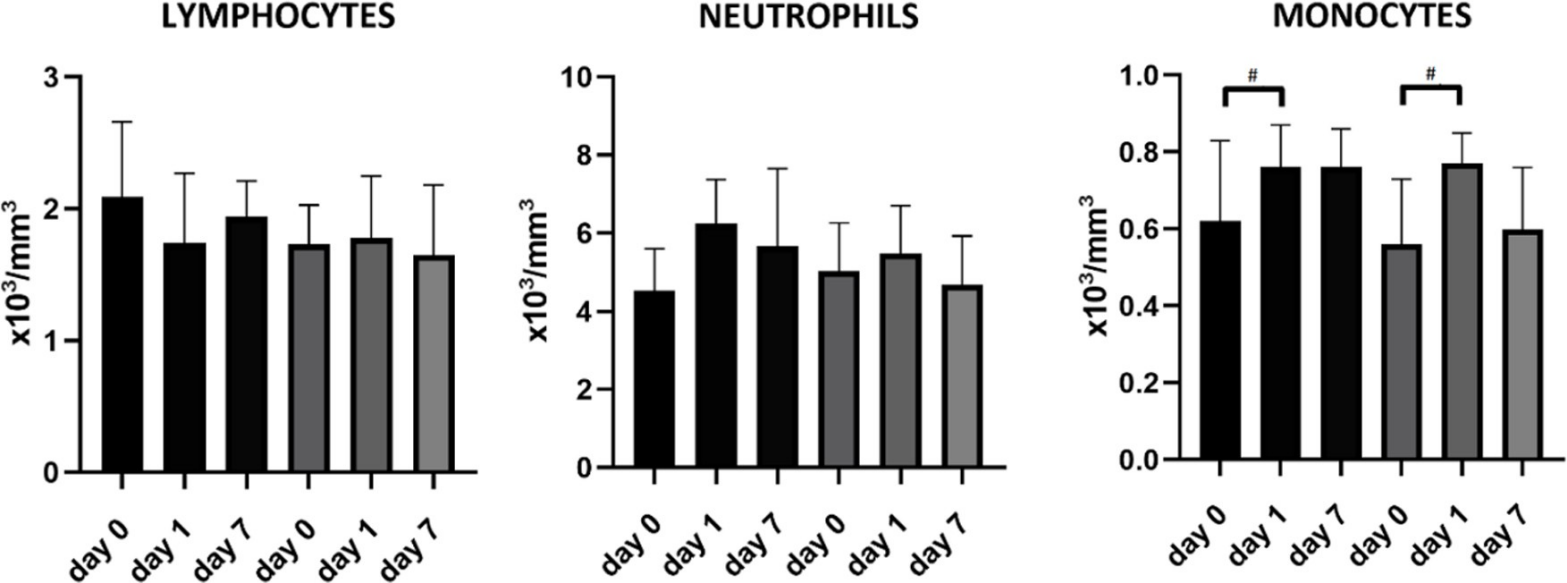
Time course of lymphocytes, monocytes and neutrophils in patients with and without spleen volume reduction, displayed as median with IQR. Patients with spleen volume reduction are shown in black, patients without in grey. In the group without spleen volume reduction, a complete dataset regarding lymphocytes, monocytes and neutrophils was missing in 8 patients. #Indicates significant difference between two different days within the same group.

Fig 1 shows the time course of lymphocytes, monocytes and neutrophils. Monocytes increased from admission to day one in both groups (p = 0,003 in the group without spleen volume reduction, p = 0,03 in the group with spleen volume reduction), without significant difference between patients with versus without spleen volume reduction. In the group with spleen volume reduction, there was a decrease in lymphocytes from admission to day one (p = 0,033), however it did not remain significant after application of the Bonferroni correction. Within the group with spleen volume reduction the amount of neutrophils, monocytes and lymphocytes at admission and on day one were similar in patients with and in those without infection.

### Lymphocyte subsets

**Fig 2:**
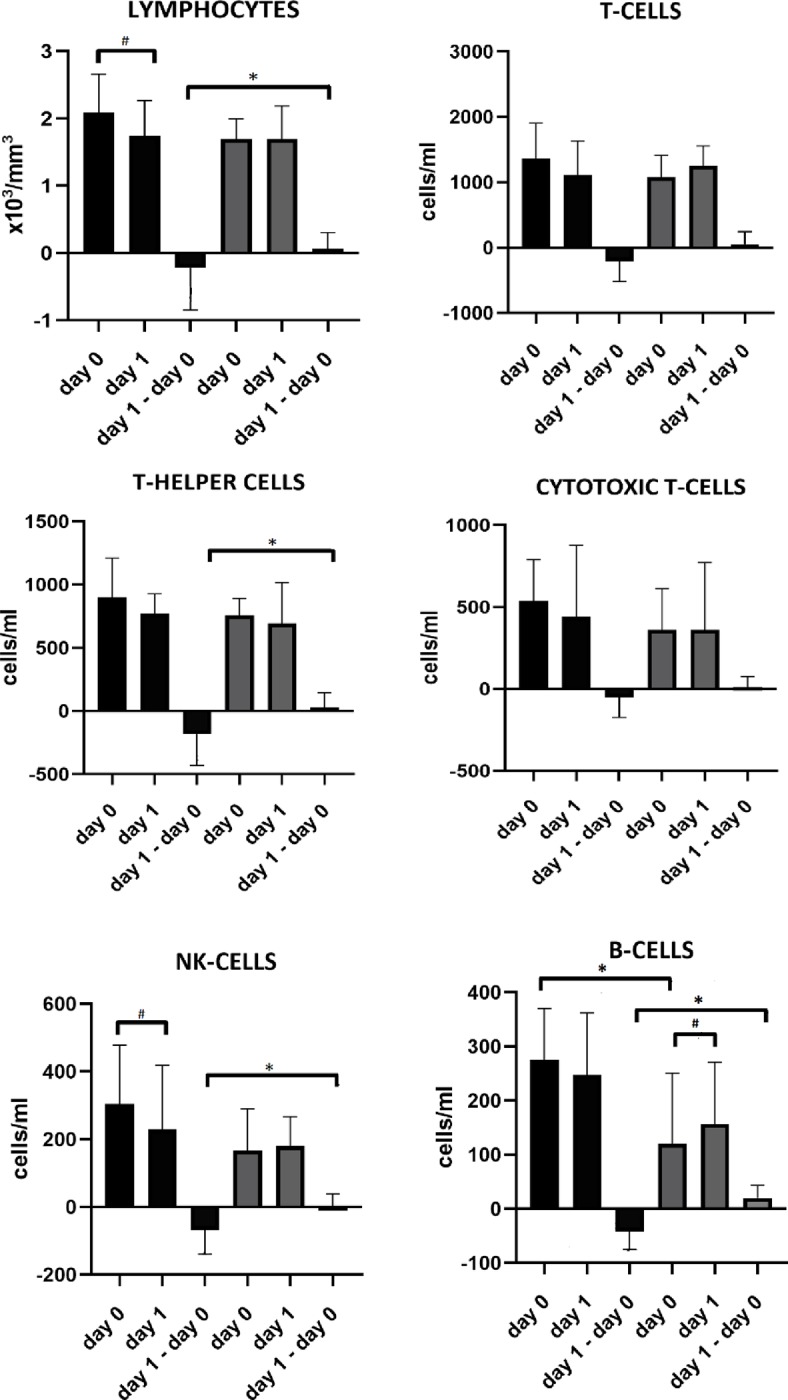
Lymphocyte and lymphocyte subset numbers on admission and day one in patients with versus without spleen volume reduction, displayed as median with IQR. Patients with spleen volume reduction are shown in black, patients without in grey. Regarding lymphocytes, 3 datasets were missing in the group without spleen volume reduction. Regarding lymphocyte subsets, 6 complete datasets were missing in the group without spleen volume reduction, 1 in the group with spleen volume reduction. # Indicates significant difference between two different days within the same group. *Indicates significant difference between both groups on the same day.

Fig 2 shows the time course of lymphocytes and lymphocyte subsets in patients with and without spleen volume reduction from admission to day one.

An increased number of B cells (275 [IQR 211; 334] cells/ml vs 114 [IQR 62;205] cells/ml, p = 0.01) on admission was found in the group with spleen volume reduction in comparison to the group without spleen volume reduction.

Patients with spleen volume reduction showed changes -mostly a drop-, in lymphocytes, T-helper cells, NK-cells and B-cells from admission to day one, which was not seen in patients without spleen volume reduction (respectively p = 0,033, p = 0,038, p = 0,022, p = 0,008). In patients with post-stroke infections (n = 11), there was a significant change, actually a drop, in lymphocytes (-0,34 x10^3^ cells/mm^3^ [IQR -0,60; -0,09] vs +0,05 x10^3^ cells/mm^3^ [IQR -0,23; +0,30] p = 0,047), T-cells (-193 cells/ml [IQR -445; -13] vs +52 cells/ml [IQR -119; +215] p = 0,043) and B-cells (-23 cells/ml [IQR -60; -11] vs +22 cells/ml [IQR +1; +40] p = 0,008) from admission to day one compared to the group without infections (n = 27). There were no significant changes in the other lymphocyte subsets. In patients with post-stroke infection (n = 11), there was an overall trend towards larger decreases in lymphocytes and lymphocyte subsets, except for cytotoxic T-cells, in the group with splenic contraction (n = 6) versus in those without (n = 5) but without statistical significance.

Within the group with spleen volume reduction, a decrease in lymphocytes (p = 0,033) and NK cells (p = 0,028) occurred between admission and day one. In the group without spleen volume reduction, there was an increase in B-cells from admission to day one (p = 0,033).

## Discussion

The main finding of our preliminary study is that in a subset of patients (29%) spleen volume reduction occurred between one and seven days after ischemic stroke and that this was associated with post-stroke infections, a higher NIHSS score on admission and at day three and with a drop of several lymphocyte subsets within the first 48 hours after admission. Both animal and human literature reported spleen volume reduction 24 to 48 hours after stroke, followed by a re-expansion, which might be linked to a loss of splenocytes and increased risk of infection. [1–4,8,9] Sahota et al, however, showed that some patients had a persistent decrease in spleen volume, which was associated with an increased risk of poor clinical outcome. They found no differences in spleen volumes for patients with and without infection. [3] However, they did not compare infection rate between patients with versus without spleen volume reduction. Vahidy et al found 40% of stroke patients to have a splenic contraction, which was associated to higher NIHSS on admission and during the course of the hospitalisation. [13] This is partially in line with our findings. However, in an extension of this study, early splenic contraction (<24 hours) was not associated with systemic inflammatory response syndrome, defined as two of the following being present: elevated heart rate, changes in body temperature, elevated respiratory rate and white blood cell changes. [14]

Spleen volume reduction was associated with an increased number of B-cells on admission and an early decrease of lymphocytes, T-helper cells, NK-cells and B-cells within the first 48 hours after admission. Earlier studies found a sustained decrease in lymphocyte subsets (B-cells, T-cells and NK-cells) in blood and spleen starting 12 hours after stroke induction in mice. [6,15] The state of immunodepression resulted in an increased susceptibility to bacterial infections and was associated to spleen atrophy.[6] Lymphocytopenia is a consistent hallmark of immunodepression in human studies. [16] Also in our study, patients with post-stroke infections had significant drops in lymphocytes, T- and B-cells from admission to day one as compared to those without infections. Zha et al found a higher percentage of lymphocytes in patients with splenic contraction [14], which is not completely in line with our findings. This might be partially explained by another definition of splenic contraction but also due to different time windows in which the blood samples were collected. The first sample was collected within the first 24 hours, and not at admission as in our study, so the early drop in lymphocytes might be missed. They did not study lymphocyte subsets.

We found a higher number of B-cells in patients with splenic contraction. We hypothesize that this might be due to a very early release of these cells from the spleen into the blood (before the apoptotic phase), as a response to inflammation in the brain. B-cells seem to play a dual role in the immune system: antibody producing subtypes protect against infection, however, interleukin-10 (IL-10) producing subtypes supress immunity. [17] On the other hand, they decrease the infiltration of neutrophils and inflammatory T-cells in the damaged brain tissue, which might have a beneficial effect on infarct growth. [18] Vahidy et al found increased levels of IL-10 and interferon gamma (INF-y) in patients with splenic contraction. [13] Our hypothesis is that in patients with more severe strokes, the spleen contracts to release B-cells, more specifically IL-10 producing B-cells as a reaction to decrease the brain damage but which might lead to immune suppression, so increased infection rate. The role of B-lymphocytes in stroke needs further research. [19] It might be interesting to study cytokine levels, such as IL-10 and INF-y in patients with and without splenic contraction, and to correlate them with lymphocyte subset numbers.

This study has several limitations: data should be interpreted with caution because the number of participants is low. Infarction volumes were small, so our results might not be representative for larger stroke volumes. We did not measure spleen volumes at admission, and cannot make a statement on very early spleen volume changes. In addition, there is no standard definition for spleen volume reduction. There is great variability in spleen volumes, not only between patients but there are also day-to-day variations within the same patient. Vahidy et al found that median spleen volumes over a five day period ranged between 122.8 cm^3^ and 133.4 cm^3^ in healthy volunteers. [13] This means that the median spleen volumes differ +/- 8,6% in a time period of five days in healthy volunteers. Within the same healthy volunteer they found day-to-day variations of 10 cm^3^. This supports our definition of a spleen volume reduction as a reduction of >10%.

Current literature suggests the role of the sympathetic nervous system in splenic contraction and post-stroke immunosuppression. [1,6,7,9,11,20–22] However, we could not find any correlations between spleen volume change and PRV parameters. This might be due to the fact that PRV parameters in resting conditions are mainly influenced by the parasympathetic nervous system, and far less by the sympathetic nervous system. [22]

## Conclusion

We found an association between spleen volume reduction between 24 hours and seven days after acute ischemic stroke and post stroke infections. This was accompanied by an early decrease in several lymphocyte subsets.

Further studies in larger study populations are needed to investigate the role of different lymphocyte subsets and to study whether the spleen is actively involved in the development of post-stroke infections or whether it is simply an innocent bystander. Also the precise role of autonomic dysfunction in the development of splenic volume reduction in acute ischemic stroke patients needs further exploration.
